# Direct assessment of ionic liquid fragility from transport property variation at moderate temperatures

**DOI:** 10.1039/d6ra03960j

**Published:** 2026-05-20

**Authors:** Adhip Rahman, Shayanta Chowdhury, Md. Abu Bin Hasan Susan

**Affiliations:** a Biochemistry and Microbiology Department, North South University Dhaka-1229 Bangladesh; b Department of Chemistry, University of Dhaka Dhaka-1000 Bangladesh susan@du.ac.bd

## Abstract

An approach to assess the ionic liquid (IL) dynamic fragility is discussed in this work; for this, viscosity data (a transport property) of 48 ILs of different classes (aprotic, protic, surfactant and magnetic) were surveyed. The fitting approach taken here enables, for the most part, IL-viscosity data at relatively mild to moderate temperatures for precise reproduction of experimentally resolved glass transition temperatures (*T*_g_). This, in turn, suggests that ILs are likely to be intermediate between strong- and highly fragile liquids – analogous to ionic inorganic melts such as ZnCl_2_, Ca(NO_3_)_2_·4H_2_O, or CKN (mixture of calcium and potassium nitrates).

## Abstract 2

An approach to assess the ionic liquid (IL) dynamic fragility is discussed in this work; for this, viscosity data (a transport property) of 48 ILs of different classes (aprotic, protic, surfactant and magnetic) were surveyed.

## Graphical abstract

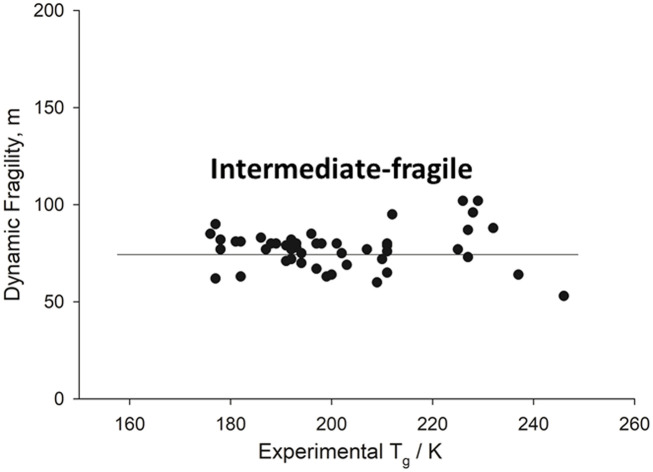

As with silicates,^1^ inorganic melts,^1,2^ and molecular liquids,^2,3^ the glass-forming ability of ionic liquids^4^ (ILs) is widely reported.^5,6^ In general, glass formers have well-defined glass transition temperatures (*T*_g_) below their crystallization points. They tend to deviate from the Arrhenius behaviour; *i.e.*, data for logarithms of transport properties (viscosity, diffusion *etc.*) against temperature diverge from linearity – hence are not fitted by the Arrhenius equation.^1–3,5,6^ Rather, three-parameter fitting models are required. The most utilized is the phenomenological Vogel–Fulcher–Tammann (VFT) equation.^2,7,8^1
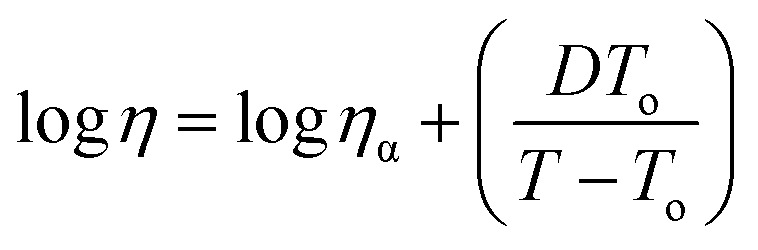


The equation here corresponds to temperature-dependent viscosities, where *η* is the viscosity at a given temperature *T*, and *D*, *T*_o_, and *η*_α_ are fitting parameters. For glass-formers, the extent of this departure is understood by “fragility”,^2,9^ which identifies a glass-former as “strong” or “fragile” as2
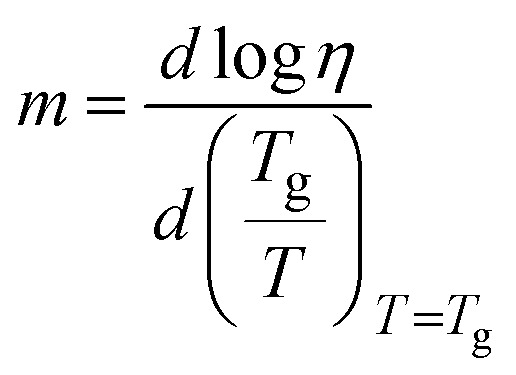


*m* is the so-called kinetic (or dynamic) fragility index. It is suggested that strong glass-formers with extensive covalent/non-covalent interatomic/molecular networks have low *m* (⪅30.^9,10^ Conversely, weaker molecular networks (observed in many organic molecular liquids) correspond to “fragile” systems with high *m* values (⪆100).^2,11^ Works related to ILs suggested that IL fragility may be intermediate between the two extrema (*m* ≈ 45–100).^5,12,13^ However, these values are far from being conclusive due to the narrow class of ILs studied in these works as well as deficiencies related to the methodologies utilized. In earlier works, the *m* values were estimated either by using data for transport properties against temperature, and subsequent VFT fitting, or from differential scanning calorimetry (DSC) thermograms.^2,13^

The VFT-fitted *m* has several issues. First, VFT is by origin a parameter-based fitting equation with little theoretical justification.^1,7,8^ The VFT-fitted data predicts the entropy for local configurational change, or configuration entropy (*S*_c_), becoming zero at a temperature *T*_o_ much higher than the absolute zero – which is unphysical.^1^ Second, for estimating *m* from the VFT-fitted *T*_g_-scaled Arrhenius plot^2^ (or so-called Angell plot), low temperature transport properties measurements close to the *T*_g_ should be made – which often is experimentally challenging. Third, the *η*_α_, or the viscosity at infinitely high temperature, in the case of VFT fitting is assumed to be 10^−4^ Poise^1^, but was not experimentally resolved.

Meanwhile, analysis of the DSC thermogram results in the “thermodynamic” fragility at the *T*_g_. However, there is no one universal DSC thermogram analysis protocol to estimate *m*; several approaches^2,10,12,14^ have been proposed. The thermochemical method is not direct as well – the *T*_g_ needs to be identified first. However, the precise detection of *T*_g_ of a material is linked to factors such as the cooling rate, annealing period, sample purity, and the thermal history;^15^ in fact, there are many ILs for which the *T*_g_ may not be correctly identified, or identified at all (for examples, see ref. 2 and 38–40 in the, SI).

The concern, therefore, is if a simpler yet efficient and direct approach can be formulated so that standard transport property (viscosity, electrical conductivity *etc.*) data at mild to moderate temperatures (such as between 273 K and ∼373 K) can be utilized to assess IL-fragility.

As part of a broader work, here we discuss the applicability of two of the most utilized parameterized models for assessing and predicting IL-dynamic fragility. Data for viscosity over temperature change for an extensive library of 48 aprotic and protic ILs with wider anionic and cationic variation (Tables S.1 and S.2 in (SI) list the ILs) were considered. Dynamic viscosity at moderate temperatures were either measured by an Ostwald falling-ball viscometer (experimental details in SI, Section S.1) or taken from literature (full list of the sources is provided in Section S.2 in SI). The fitting involves the VFT equation [see eqn (1)] and the thermodynamically more “reasonable” MYEGA (Mauro–Yue–Ellison–Gupta–Allan) equation as introduced by Mauro *et al.*^1^3
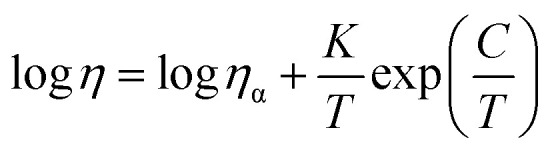
In the MYEGA equation, *K* and *C* represent the change of entropy related to the local molecular/atomic configuration states upon changing temperature. The MYEGA equation takes the thermodynamic assumptions of the Adam–Gibbs equation^16^ and extends this in that the entropy of atomic/molecular configuration change (*S*_c_) accounts for a correlated cluster of atoms/molecules (the constraint in the local configuration arises from the covalent bond/noncovalent network) surpassing a potential energy barrier – leading to transport properties variation upon temperature change.^1,17,18^ As such, the fitting parameters of the MYEGA equation have some theoretical basis unlike the VFT equation.

We fit log *η vs. T* and log *η vs.* 1/*T* plots (Fig. S.1 in SI for a representative series of ILs) by means of the following two modified forms of the original (eqn (1) and (3)) VFT and MYEGA equations (for details related to the derivation of these equations, see SI) respectively.^1^4
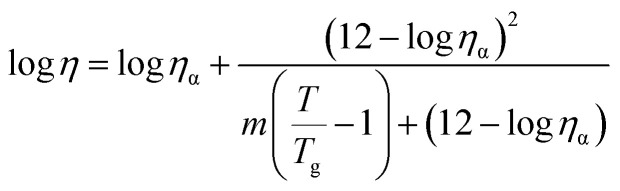
and,5
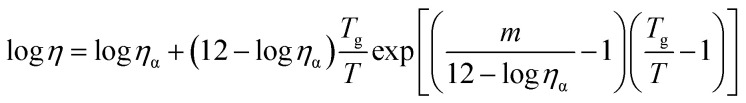


In these forms, the viscosity at *T*_g_ of network glass-formers was assumed to be 10^12^ Pa s,^1^ and *m* is defined by eqn (2). Eqn (4) and (5) contain *m*, *T*_g_ and *η*_α_ as the adjustable parameters (for context, the basic forms do not contain *m* and *T*_g_ among the adjustable parameters). This is why these forms were utilized for the fitting purposes. However, the *η*_α_ was not left as a floating parameter. Instead, specific *η*_α_ values based on analyses by Zheng *et al.*^19^ were used – for all MYEGA fits the “universal” *η*_α_ was taken as 10^−2.9^ Pa s; for the VFT fits *η*_α_ was considered as 10^−3.9^ Pa s. In both cases, the standard deviation (s.d.) was considered as s.d. ≈ 0.3.^19^ Specifically, it was shown^19^ that while the MYEGA fitted *η*_α_ = 10^−2.9^ Pa s agrees viscosity data best the covalent-network glasses, it broadly agrees to certain alkylimidazolium and chloroaluminate-based ILs too.

As an example, the fitted mean *m* and *T*_g_s are shown against experimentally resolved values (Table S.3, SI) in Fig. 1 for a series of aprotic 1-alkyl-3-methylimidazolium ILs.

**Fig. 1:**
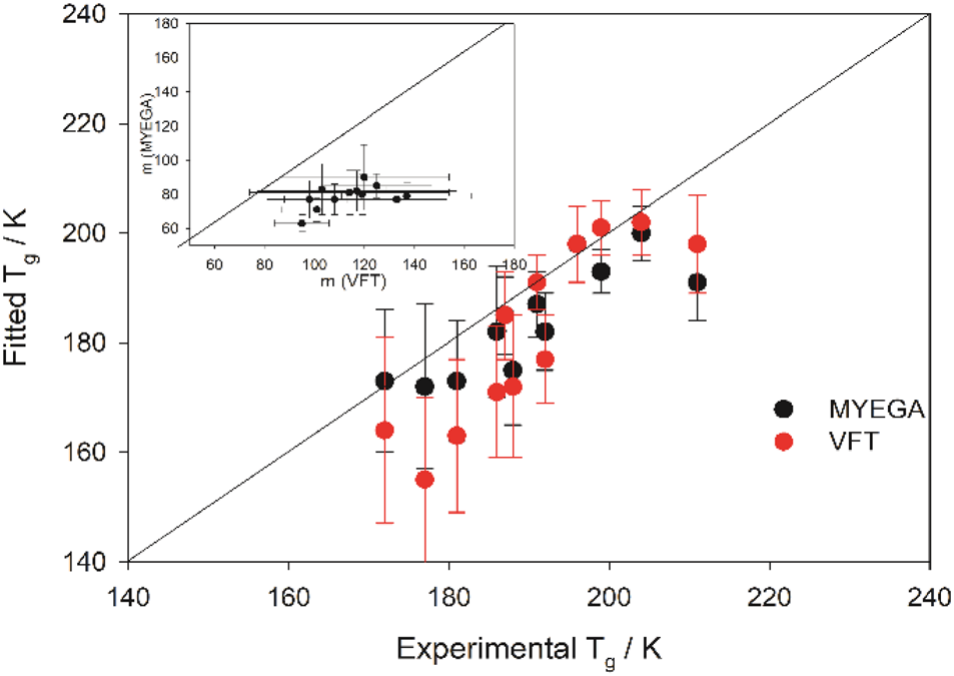
Comparison of experimental *T*_g_s with modified-MYEGA (at *η*_α_ = 10^−2.9±0.3^ Pa s) and modified-VFT (at *η*_α_ = 10^−3.9±0.3^ Pa s) fitted mean *T*_g_s of 1-alkyl-3-methylimidazolium ILs listed in Table S.3; experimental *T*_g_s were taken from literature (see Table S.2, SI). The inset shows correlation between the modified MYEGA and VFT-fitted mean m values. The “error bars” represent a simplistic standard deviation from the mean values, see SI for more details. The lines are guides to the eye.

The values corresponding to these, and all the other ILs surveyed, are listed in Table S.3 in the SI (also see section S.1 for more details).

From these data, certain aspects can readily be observed – (i) the modified VFT predicts the *T*_g_s reasonably well for majority of the ILs (Table S.3), but unusually high IL-fragility with “wider” s.d. (to note, the s.d. values corresponding to the *m* and *T*_g_s resulted from the two extrema of the *η*_α_ standard deviation window considered in this work, see above, and SI for more details). While the dynamic fragility *m* estimated from high-temperature transport data often appeared to be slightly higher (*m* ≈ 75–90) than the thermodynamic fragility,^11–14^ the VFT-predicted *m*-fragilities estimated for some of the ILs in this work are clearly unreasonable (m as high as 120 and above, see Table S.3) given long-range electrostatic forces and interionic H-bonds are commonplace in ILs^20–22^ – due to which ILs and ionic glass formers show an inverse correlation between non-exponential structural relaxation and fragility.^23^ This trend is unlike the highly fragile molecular glass formers in which the structural relaxation markedly deviates^9^ from a single exponential decay. In this sense, the long-range electrostatic network strength, and, in turn, *m* fragility, of ILs should be comparable to many metallosilicate glasses,^23^ inorganic melts like zinc chloride (ZnCl_2_),^24^ and/or, to the very least, strong H-bonding network liquids^14,25–27^ like glycerol. The modified-VFT fitted *m* fragilities do not reflect this. To further note, the fitting efficiency of the basic VFT form (eqn (1)) was also tested. The liquid-specific *η*_α_ = 10^−5^ Pa s, as suggested by Angell,^28^ was utilized, and the fragilities were calculated according to the following equation.^29^6
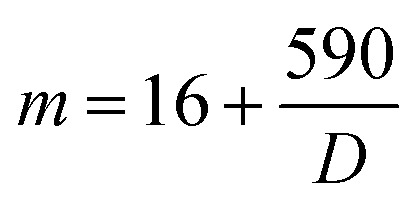


The fragilities of some commonplace ILs are compiled and compared in Table 1. According to the original VFT form, *m*-fragilities of [C_2_mim]BF_4_ and [C_2_mim]NTF_2_ are quite low, and they do not agree well with the dielectric-derived, VFT-fitted *m*-fragilities for the same ILs.^24,25^ Nevertheless, for the other ILs, the VFT-estimated (both forms, eqn (1) and (4)) *m*-fragilities were mostly around or over 100. It should be noted that the modified VFT-fitted *m* values appear much below 100 if *η*_α_ < 10^−3.9^ Pa s; however, in such a case, the fitted *T*_g_s diverge significantly from the experimentally resolved *T*_g_s (Tables S.2 and S.3, SI) – regardless the IL chemical nature.

**Table 1:** Comparison of VFT and MYEGA-fitted dynamic fragilities (*m*), and glass transition temperatures (*T*_g_) of selected ILs

ILs	*m* a	Fitted^a^*T*_g_/*K*	Experimental^b^*T*_g_/*K*
[C_2_mim]NTf_2_	81	172	181^c^
	113	165	
	49	—	
[C_2_mim]MeSO_3_	79	191	211
	137	198	
	102	—	
[C_2_mim]OAc	85	197	196^c^
	158	198	
	129	—	
[C_2_mim]BF_4_	82	173	178
	113	164	
	51	—	
[C_4_mim]BF_4_	77	185	187
	108	185	
	110	—	
[C_8_mim]BF_4_	72	190	190^c^
	119	199	
	114	—	

a Values for each of the ILs are organized in the following order (top to bottom): modified MYEGA (eqn (4)), modified VFT (eqn (5)), and VFT (eqn (1)); fitted values correspond to fits at *η*_α_ = 10^−2.9^ Pa s (modified MYEGA) and *η*_α_ = 10^−3.9^ Pa s (modified VFT).

b Taken from literature; for the full list of references, see Table S.2, SI.

c Multiple *T*_g_s reported in the literature, for multiple *T*_g_s and the full list, see Table S.3, SI.

On the other hand, the modified MYEGA equation shows better efficiency in predicting the *T*_g_s as well as better assessing the *m* values. Table 1, in addition to the VFT-fitted values, collates the MYEGA-fitted *m* and *T*_g_s of these ILs (the full list can be found in Table S.3). The MYEGA-predicted *m*-fragilities are much lower compared to the corresponding VFT-fitted ones. Moreover, the MYEGA predicted fragilities agree very well with the *m* windows suggested for many “ionic” glass formers (examples: ILs, inorganic oxide, nitrate, and halide melts^2,3,9,12,13,24,26^).

As such, our key focus is on the MYEGA fitting henceforth. The fitting proposed here involves calibration of the *η*_α_ such that the fitted *T*_g_ approaches as close as possible to the experimentally resolved *T*_g_s of the ILs with a lesser s.d. Subsequently, the s.d. values to these *m*-fragilities too would be lesser. For MYEGA-fits, the convergence of the fitted and experimental *T*_g_s becomes increasingly precise when the lower end of the *η*_α_ s.d. is considered (*i.e.*, *η*_α_ between 10^−2.6^ and 10^−2.9^ Pa s); this is in line with what Zheng *et al.*^19^ observed for metallic, and ionic glasses.

Therefore, for a second batch trial we consider, *η*_α_ = 10^−2.7±0.2^ Pa s. The newly fitted *T*_g_s (Table S.3) approach further close to, if not precisely overlap, the experimental *T*_g_s – for the majority of the ILs (except some hydrogen sulfate anion containing ILs, see below) surveyed for this work.

To better discern the MYEGA equation's efficacy in assessing the IL dynamic fragility, in Table 2 we closely analyse the *T*_g_s and *m* of a number of aprotic and protic ILs having the triflimide [NTf_2_]^−^ ion at *η*_α_ = 10^−2.7^ Pa s. We find that the bulkier the cation, the stronger is the IL, *i.e.*, *m* decreases – suggesting strengthened interionic networks. This trend is strongly supported by dielectric relaxation data^13^ on IL-fragility relation to the alkyl-chain length of imidazolium ILs. An advantage of the approach reported in this work is that viscosity data at mild or moderate temperatures estimate the fragilities to the same extent to the low-temperature dielectric data. Meanwhile, recent neutron diffraction experiments^30^ suggested that the hydrogen bonding involving the cationic proton and the anionic O atoms gets stronger when the cation becomes bulkier from [C_2_mim]^+^ to [C_10_mim]^+^ (Table S.1 for the structures). The trend can be noticed in ILs containing other anions, such as tetrafluoroborate [BF_4_]^−^ and ethyl sulfate [EtSO_4_]^−^ (Table S.3). The VFT-fitted data did not show such trend (Table S.3). The surfactant ILs (SILs)^30,31^ are of particular interest too – we fitted dynamic viscosity data (for ref. see Table S.2) for SILs containing the bis(2-ethylhexyl)sulfosuccinate anion from the surfactant Aerosol-OT.^30^ The *m* values (Table S.3) are between 60–70 – suggesting stronger network than shorter alkyl-chain ILs. This is reasonable given that these SAILs exhibit ordered, self-assembly in the bulk.^30,31^

**Table 2:** Comparison of the MYEGA-fitted *T*_g_s to experimental *T*_g_s and respective fragility indices (*m*) of [NTf_2_]^−^ ILs

ILs	Experimental *T*_g_^a^/*K*	Fitted *T*_g_ and *m* at *η*_α_ = 10^−2.7^ Pa s
[C_2_mim]NTf_2_	186	185, 94
[C_4_mim]NTf_2_	186	189, 92
[C_6_mim]NTf_2_	192	191, 91
[C_8_mim]NTf_2_	193	192, 86
[C_4_mPy]NTf_2_	189	188, 88
[C_6_mPy]NTf_2_	191	190, 87
[C_8_mPy]NTf_2_	193	193, 86

a Taken from literature; for the full references list, see Table S.2 in SI.

Another striking aspect involves the proton donating and accepting hydrogen sulfate^29^ HSO_4_^−^ ILs. Because these ILs induce a much stronger H-bond network by means of Grotthuss-type^32^ ionic transport, they should have relatively low *m*-fragilities. Ueno *et al.*^29^ showed the thermodynamic fragility of decahydroisoquinoline-based ([DHiQ]HSO_4_) IL to be 45. We performed a MYEGA fit over their viscosity data, which gives the *m* ≈ 53 ± 5 (at *η*_α_ = 10^−2.9±0.3^ Pa s) – close enough to their prediction. For this particular IL, however, the fitted *T*_g_ did not converge well with the experimental *T*_g_ (Table S.3), although this issue was not faced for the other HSO_4_^−^ ILs we surveyed (note, the DSC thermograms of these HSO_4_^−^ ILs were analysed by two different protocols^29,33^). Nevertheless, the *T*_g_s predicted by the MYEGA agree well for most of the protic ILs (PILs) listed in this work, but the *m*-fragilities are on the higher end (there are certain PILs for which the viscosity-fitted *T*_g_s appeared to be somewhat higher than their experimental *T*_g_s, see Table S.3, SI. The reason cannot be ascertained at this point). For the DHiQ ILs, the *m* values are much higher compared to the corresponding thermodynamic fragilities.^29^ This is counterintuitive as one would imagine the PILs having stronger H-bond networks than the aprotic ILs (AILs) should be stronger glass formers. One reason may be the potential water uptake by PILs. Another possibility is a potential fragile-to-strong (FTS)^24^ transition in these ILs, *i.e.*, at moderate temperatures, the viscosity data results in higher dynamic *m*-fragilities that may gradually decrease as the temperature lowers towards the *T*_g_. For example,^24,25^ the ionic melt ZnCl_2_ shows similar behaviour; the MYEGA-fitted ZnCl_2_ viscosity data gives a dynamic fragility *m* = 59, meanwhile, calorimetric measurements give a thermodynamic fragility *m* = 30. The scenario may be reasonable to assume as correlated local heterogeneity (*i.e.*, nanosegregation, ion-cluster formation *etc.*) in ILs is well-known.^30,34,35^ However, structural relaxation studies are needed to confirm this phenomenon in these ILs.

In Fig. 2, we re-assess the correlation between the (MYEGA-derived) dynamic fragility *m* and *T*_g_; Qin and McKenna^26^ compiled data and showed that the correlation is strongly dependent on the material property. For example, a linear increase of *m* on increasing *T*_g_ was benchmark for H-bonded organic liquids, polymers, and metallic glasses. No such correlation was found for inorganic network glass-formers (borosilicates, CAS, Na_2_O·SiO_2_ fuses *etc.*); for ionic glass formers, no conclusion could be drawn due to limited data. It is noteworthy that according to Fig. 2, ILs are broadly akin to inorganic network glasses^26^ in terms of this correlation.

**Fig. 2:**
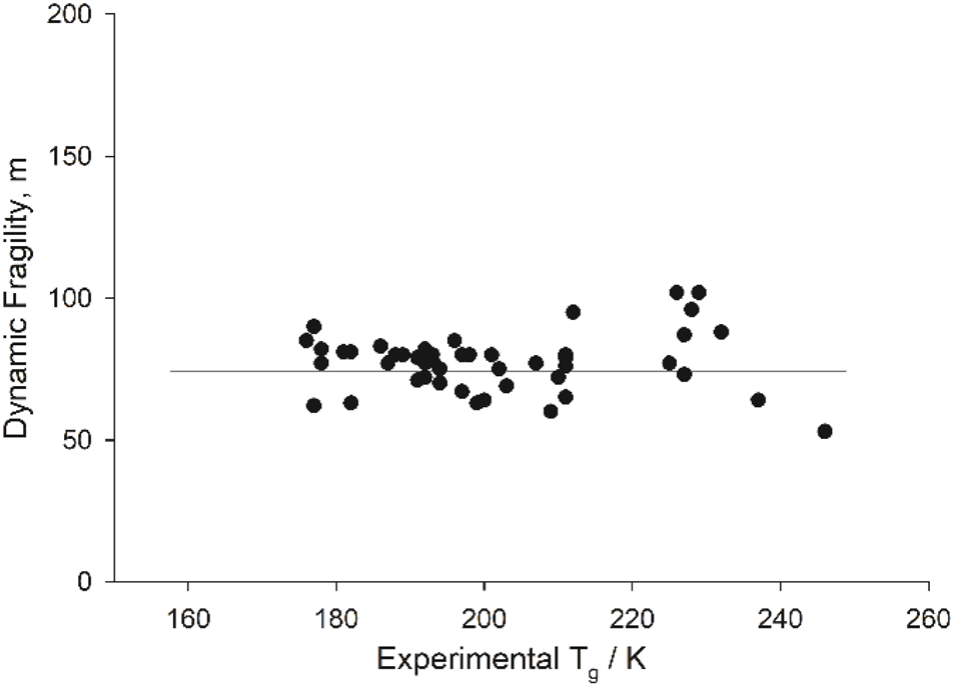
Correlation between MYEGA-derived mean of m (*η*_α_ = 10^−2.9^ Pa s) and experimental *T*_g_s of the ILs surveyed in Table S.3 in the SI. The line is a guide to the eye.

There are some limitations of the model-fitting approaches discussed in this work, however. One such case has been mentioned above – regarding the *T*_g_ of certain [DHiQ]^+^ ILs. Another issue is that this approach may not address the potential liquid-to-liquid (LL) transition^36^ above the *T*_g_s of certain ILs. For instance, DSC measurements suggested that the trihexyltetradecylphosphonium IL [P_666,14_]NTf_2_ may show an LL transition at around 201 K and a *T*_g_ at 195 K.^36^ However, MYEGA-fit of limited viscosity data (283–315 K, see ref. 37 in the SI) of the same IL suggested a fitted *T*_g_ ≈ 180 K – potentially contradicting the experiment.^36^ The narrow temperature window, hence limited number of viscosity datapoints, may be the potential reason. Interestingly, when the inverse of dc-conductivity (*i.e.*, dc-resistivity, *σ*_dc_^−1^) data^36^ against temperature (273–373 K) for the same IL were fitted by the MYEGA equation, a *T*_g_ = 197 K and *m* ≈ 53 was obtained (details about the adaptation of the MYEGA equation for dc-resistivity is given in the SI). It is too early to comment on the m-fragility obtained from the dc-resistivity fit – as, similar to *η*_α_, optimization of a “universal” dc-resistivity at infinite temperature (*σ*_α_^−1^) is required. This is one of our future interests. Nevertheless, it is reasonable to believe that data for a wider temperature-window, even in the mild-to-moderate temperature regions, may induce more precise fitted-*T*_g_s.

In short, this work suggests that the MYEGA model may be more efficient and direct in assessing IL dynamic fragility from viscosity data at moderate temperatures. In our appraoch, the goal was to match the fitted *T*_g_-values to the experimentally resolved *T*_g_s, which in turn would provide the dynamic fragility index *m* as the other floating parameter. The key to the fitted-*T*_g_s precisely overlapping with experimental *T*_g_s was optimizing the *η*_α_; based on earlier predictions and the extensive survey of ILs in this work, the optimal *η*_α_ is suggested to be roughly 10^−2.6^–10^−2.7^ Pa s. While the VFT fitted *T*_g_s at *η*_α_ = 10^−3.9^ Pa s agree well to experimentally resolved values, the limitations in assessing dynamic fragilities were quite evident. The MYEGA-fitted *m* values suggest the obvious^5,12,13,26,29^ that ILs are intermediate-to-moderately fragile liquids (*m* ≈ 50–90), and are akin to ionic inorganic melts.

## Author contributions

A. R.: experiment, data curation, literature survey, analysis, writing, editing, reviewing; S. C: data curation, literature survey, analysis, editing, reviewing, M. A. B. H. S.: project acquisition, writing, editing, reviewing.

## Conflicts of interest

There are no conflicts to declare.

## Supplementary Material

RA-016-D6RA03960J-s001

## Data Availability

The data supporting this article have been included as part of the supplementary information (SI). Supplementary information: experimental details, data for viscosity over temperature, chemical structures of the ILs surveyed, full reference list for the sources of additional viscosity and glass-transition data, and full list of the fitted-m and -*T*_g_ values. See DOI: https://doi.org/10.1039/d6ra03960j.
