# Supplementary material for: Direct assessment of ionic liquid fragility from transport property variation at moderate temperatures
Source: RSC Adv. 2026 May 20;16(29):27085–9. doi: 10.1039/d6ra03960j (PMC13191757; doi:10.1039/d6ra03960j)
Supplement: RA-016-D6RA03960J-s001 [file RA-016-D6RA03960J-s001.pdf]

Supplementary Information (SI) for RSC Advances.
This journal is © The Royal Society of Chemistry 2026

Supplementary Information

Direct assessment of ionic liquid fragility from transport property variation at moderate temperatures

Adhip Rahman[a], Shayanta Chowdhury[b§], and Md. Abu Bin Hasan Susan[b*]

[a]*Biochemistry and Microbiology Department, North South University, Dhaka – 1229, Bangladesh*

[b]*Department of Chemistry, University of Dhaka, Dhaka – 1000, Bangladesh*

*Email correspondence: susan@du.ac.bd

[§]*Current Address: Dept. of Chemistry and Biochemistry, University of Notre Dame, IN 46556, USA.*

S.1. Experimental Details

Chemicals

The five aprotic ILs used for viscosity measurement (see Table S.2 below) were obtained from Sigma-Aldrich, and were used as received (water content < 100 ppm).

Viscosity measurement

Dynamic viscosities of the samples were obtained by using an Anton Paar Lovis 2000 ME rolling-ball viscometer (precision level, ±0.001 mPa s) within a temperature range of 293-343 K (precision level, ±0.01 K) at 5 K intervals.

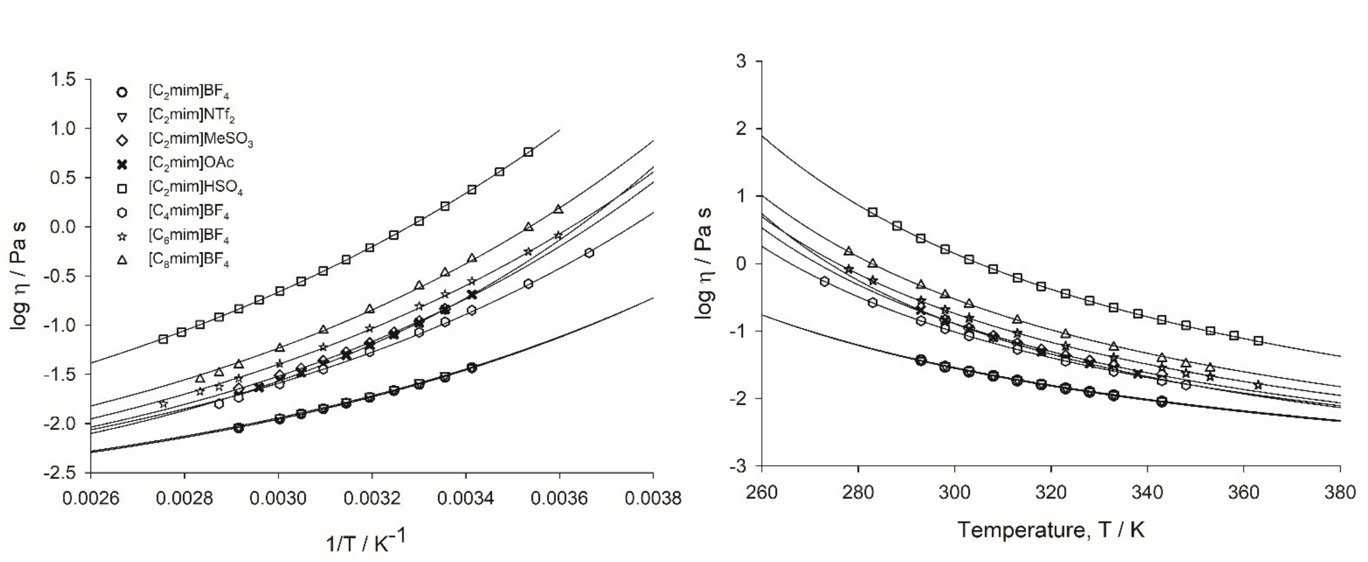

Figure S.1. Dynamic viscosity values on increasing temperature for a representative series of ILs and fits (solid lines) to - (left) modified MYEGA, and (right) modified VFT equations. Data for [C_2mim]BF_4, [C_2mim]NTf_2, [C_2mim]OAc, and [C_2mim]$MeSO_3$ are given in Table S.4, data for the rest of the ILs were taken from the references listed in Table S.2 (see below).

Table S.1. Chemical structures of the cations and anions of the ILs surveyed in this work.

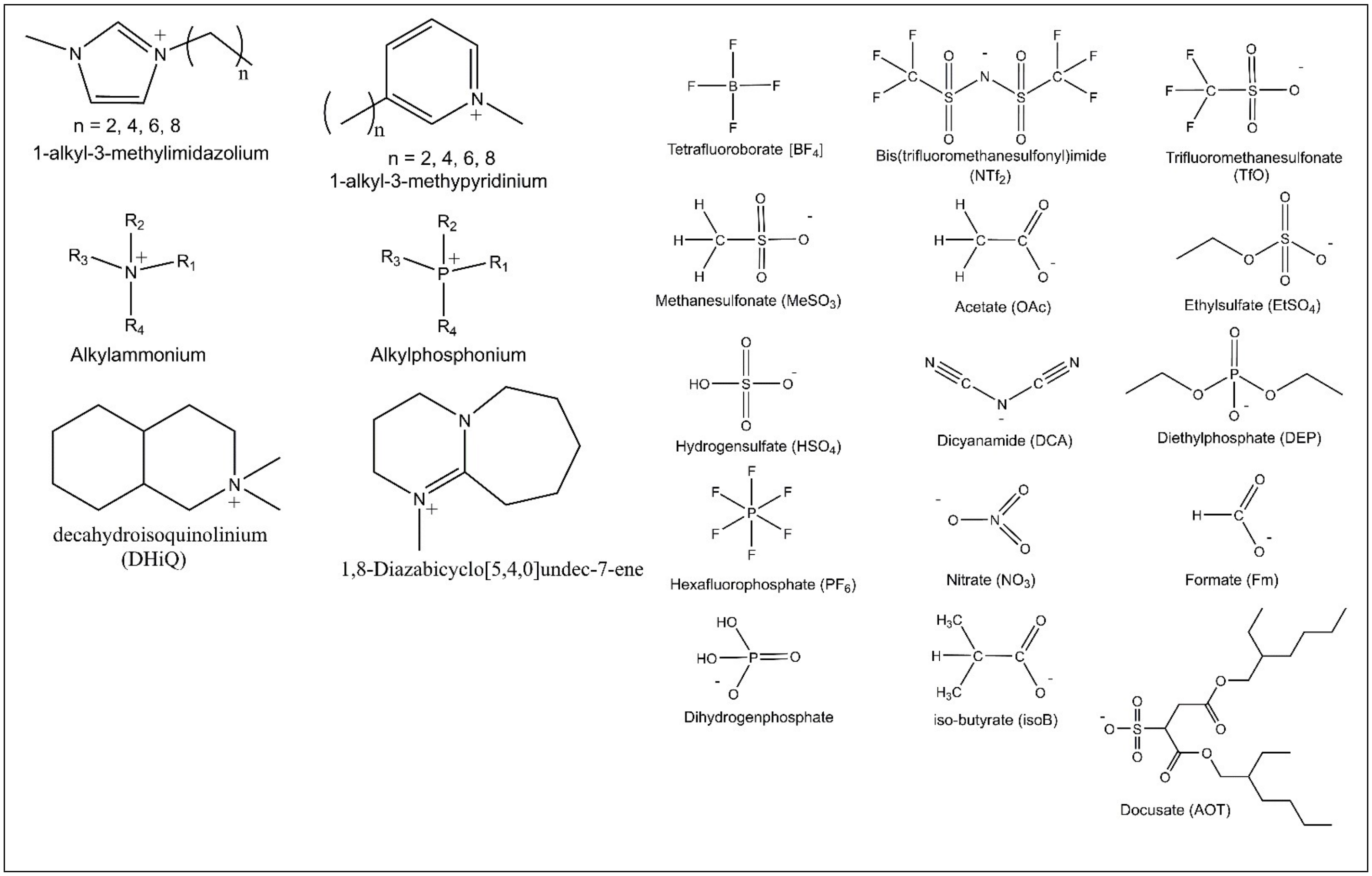

Table S.2. List of ILs surveyed in this work, references from which T_gs, and viscosity data were taken; the full reference list can be found in the references section of the SI (see below).

ILs	Refs. for T_gs	Refs. for viscosity data
1-ethyl-3-methylimidazolium tetrafluoroborate, $[C_2mim]BF_4$	[1]	this work
1-ethyl-3-methylimidazolium bis(trifluoromethanesulfonyl)imide, $[C_2mim]NTf_2$	[2], [3], [4]	this work, [2], [25]
1-ethyl-3-methylimidazolium trifluoromethanesulfonate, $[C_2mim]TfO$	[5]	this work
1-ethyl-3-methylimidazolium acetate, $[C_2mim]OAc$	[6], [7], [8]	this work
1-ethyl-3-methylimidazolium methanesulfonate, $[C_2mim]MeSO_3$	[9], [16]	this work
1-ethyl-3-methylimidazolium ethylsulfate, $[C_2mim]EtSO_4$	[9],[10]	[11]
1-ethyl-3-methylimidazolium dicyanamide, $[C_2mim]DCA$	[12]	[13]
1-ethyl-3-methylimidazolium hydrogensulfate, $[C_2mim]HSO_4$	[14]	[15]
1-ethyl-3-methylimidazolium diethylphosphate, $[C_2mim]DEP$	[16], [17]	[18]
1-butyl-3-methylimidazolium tetrafluoroborate, $[C_4mim]BF_4$	[19], [20]	[21]
1-hexyl-3-methylimidazolium tetrafluoroborate, $[C_6mim]BF_4$	[22], [25]	[23]
1-octyl-3-methylimidazolium tetrafluoroborate, $[C_8mim]BF_4$	[22], [25]	[24]
1-butyl-3-methylimidazolium bis(trifluoromethanesulfonyl)imide $[C_4mim]NTf_2$	[2]	[2]
1-hexyl-3-methylimidazolium bis(trifluoromethanesulfonyl)imide $[C_6mim]NTf_2$	[2]	[2]
1-octyl-3-methylimidazolium bis(trifluoromethanesulfonyl)imide $[C_8mim]NTf_2$	[2], [25]	[2]
1-butyl-3-methylimidazolium hexafluorophosphate, $[C_4mim]PF_6$	[26]	[27]
1-octyl-3-methylimidazolium phosphate, $[C_8mim]PF_6$	[24]	[28]
1-butyl-3-methylimidazolium tetrachloroferrate, $[C_4mim]FeCl_4$	[29]	[29]
1-butyl-3-methylpyridinium tetrafluoroborate, $[C_4mPy]BF_4$	[25]	[25]
1-butyl-3-methylpyridinium bis(trifluoromethanesulfonyl)imide, $[C_4mPy]NTf_2$	[25]	[25]
1-hexyl-3-methylpyridinium bis(trifluoromethanesulfonyl)imide $[C_6mPy]NTf_2$	[25]	[25]
1-octyl-3-methylpyridinium bis(trifluoromethanesulfonyl)imide, $[C_8mPy]NTf_2$	[25]	[25]
1-ethyl-3-methylpyridinium ethylsulfate, $[C_2mPy]EtSO_4$	[25]	[25]
1-ethylnicotinium ethylsulfate, $[Et_2Nic]EtSO_4$	[25]	[25]
1-butyl-3-methylimidazolium docusate, $[C_4mim]AOT$	[30]	[31]
Tetrabutylammonium docusate, $[N_{444,4}]AOT$	[25]	[25]
Heptyltrimethylphosphonium docusate, $[P_{444,7}]AOT$	[32]	[32]
Butyltrimethylammonium bis(trifluoromethanesulfonyl)imide, $[N_{411,1}]NTf_2$	[33]	[34]
Tributylmethylammonium bis(trifluoromethanesulfonyl)imide, $[N_{444,1}]NTf_2$	[35]	[34]
Trihexyltetradecylphosphonium bis(trifluoromethanesulfonyl)imide, $[P_{666,14}]NTf_2$	[36]	[37]
Triethylammonium methanesulfonate, $[N_{222,o}]MeSO_3$	[38]	[38]
Trimethylammonium dihydrogenphosphate, $[N_{111,0}]H_2PO_4$	[38]	[38]
Ethylammonium hydrogensulfate, $[N_{200,0}]HSO_4$	[38]	[38]
Butylammonium hydrogensulfate, $[N_{400,0}]HSO_4$	[38]	[38]
Ethylammonium nitrate, $[N_{200,0}]NO_3$	[38]	[38]
Decahydroisoquinolinium bis(trifluoromethanesulfonyl)imide, $[DHiQ]NTf_2$	[39]	[39]
Decahydroisoquinolinium isobutyrate, [DHiQ]isoB	[39]	[39]
Decahydroisoquinolinium formate, [DHiQ]Fm	[39]	[39]
Decahydroisoquinolinium hydrogensulfate, $[DHiQ]HSO_4$	[39]	[39]
Decahydroisoquinolinium methanesulfonate, $[DHiQ]MeSO_3$	[39]	[39]
1,8-diazabicyclo-[5,4,0]-undec-7-ene trifluoromethanesulfonate,	[40]	[40]

[DBU]TfO		
1,8-diazabicyclo-[5,4,0]-undec-7-ene acetate, [DBU]OAc	[40]	[40]
1,8-diazabicyclo-[5,4,0]-undec-7-ene methanesulfonate, [DBU]MeSO_3	[40]	[40]
1-methylpyrinidium hydrogensulfate, [C_1Py]HSO_4	[41]	[41]
1-methylpyrrolidinium hydrogensulfate, [C_1Py]HSO_4	[41]	[41]
1-methylimidazolium hydrogensulfate, [C_0mim]HSO_4	[41]	[41]
1-butyl-3-methylimidazolium chloride, [C_4mim]Cl	[42]	[43]
1-octyl-3-methylimidazolium chloride, [C_8mim]Cl	[44]	[45]

Table S.3. Modified MYEGA- and modified VFT-fitted mean T_gs and dynamic fragilities (m) (experimental T_gs listed for comparison purposes, see Table S.2 for their sources)

ILs	Experimental T_g / K	MYEGA-Fitted T_g and m at $\eta_\alpha = 10^{-2.9\pm0.3}$ Pa s	MYEGA-fitted T_g and m at $\eta_\alpha = 10^{-2.7\pm0.2}$ Pa s	VFT-fitted T_g and m at $\eta_\alpha = 10^{-3.9\pm0.3}$ Pa s
$[C_2mim]BF_4$	178	173±13, 82±13	184±10, 95±12	164±16, 113±32
$[C_2mim]NTf_2$	170, 181, 186	172±11, 81±12	183±10, 93±12	165±14, 117±40
$[C_2mim]TfO$	180	171±10, 77±10	180±8, 87±10	164±13, 108±25
$[C_2mim]OAc$	193, 196, 207	197±7, 85±8	203±6, 93±8	207±8, 158±26
$[C_2mim]MeSO_3$	211	191±7, 79±8	198±6, 87±8	198±8, 137±27
$[C_2mim]EtSO_4$	189, 192	182±7, 77±11	188±5, 83±7	177±8, 98±17
$[C_2mim]DCA$	177	172±15, 90±17	184±12, 107±17	155±15, 120±34
$[C_2mim]HSO_4$	199	193±4, 63±4	197±3, 66±4	201±5, 95±11
$[C_2mim]DEP$	204, 207	200±5, 77±7	204±4, 83±6	209±7, 133±20
$[C_4mim]BF_4$	187	185±7, 77±9	189±6, 83±8	185±8, 108±20
$[C_6mim]BF_4$	188, 194	189±7, 75±8	192±4, 79±5	190±7, 104±18
$[C_8mim]BF_4$	190, 192	190±5, 72±6	195±4, 77±5	199±5, 119±16
$[C_4mim]NTf_2$	186	182±12, 83±15	190±9, 92±10	171±12, 103±26
$[C_6mim]NTf_2$	192	1[85±8, 82±11	184±5, 91±9	190±10, 139±29
$[C_8mim]NTf_2$	189, 193	187±8, 80±10	192±6, 86±8	192±9, 135±26
$[C_4mim]PF_6$	191	184±6, 71±7	193±3, 78±3	191±5, 101±15
$[C_8mim]PF_6$	194	195±5, 70±7 [a]	198±3, 74±4	199±6, 99±14
$[C_4mim][FeCl_4]$	188	175±10, 80±12	183±8, 90±12	172±13, 119±29
$[C_4mPy]BF_4$	197	194±5, 80±8	199±4, 87±6	198±5, 119±17
$[C_4mPy]NTf_2$	189	181±10, 80±13	188±8, 88±8	173±11, 101±24
$[C_6mPy]NTf_2$	191	184±7, 79±9 [a]	190±5, 87±6	180±8, 105±19
$[C_8mPy]NTf_2$	193	187±5, 78±9	193±5, 86±7	193±8, 134±23
$[C_2mPy]EtSO_4$	202	214±3, 75±5	217±2, 79±4	223±4, 118±12
$[Et_2Nic]EtSO_4$	211	214±2, 76±5	218±1, 82±2	223±4, 117±13
$[C_4mim]AOT$	209	196±4, 60±4	199±4, 62±4	197±4, 77±9
$[N_{444,4}]AOT$	211	208±3, 65±4	214±2, 69±3	220±4, 94±10

$[P_{444,7}]$AOT	197	203±3, 67±4	205±3, 70±3	209±4, 96±10
$[N_{411,1}]NTf_2$	193, 198	188±8, 80±9	194±6, 88±8	195±8, 139±26
$[N_{444,1}]NTf_2$	201	204±4, 80±7 [a]	208±3, 85±4	210±4, 122±15
$[P_{666,14}]NTf_2$	195	175±5, 63±5[b]	180±4, 67±4[b]	170±6, 76±11[b]
$[N_{222,0}]MeSO_3$	176	195±9, 85±11	203±7, 96±10	199±10, 145±32
$[N_{111,0}]H_2PO_4$	237	225±3, 64±3	227±2, 67±2	236±4, 100±10
$[N_{200,0}]HSO_4$	177	180±6, 62±7	186±5, 68±4	180±8, 88±15
$[N_{400,0}]HSO_4$	210	207±4, 72±5	210±3, 75±4	219±5, 121±14
$[N_{200,0}]NO_3$	182	175±12, 81±13	184±8, 92±11	169±14, 118±30
$[DHiQ]NTf_2$	227	221±4, 87±8	225±3, 94±6	227±5, 138±16
[DHiQ]isoB	226	237±3, 102±7	240±2, 107±6	--[c]
[DHiQ]Fm	212	225±4, 95±7	228±2, 101±6	--[c]
$[DHiQ]HSO_4$	246	194±7, 53±5	200±5, 57±4	195±10, 69±11
$[DHiQ]MeSO_3$	228	243±3, 96±5	246±3, 90±7	261±3, 185±20
$[C_1Py]HSO_4$	203	197±6, 69±6	202±4, 74±5	206±6, 112±15
$[C_1Pyr]HSO_4$	177	172±8, 62±6	179±6, 67±6	169±11, 83±17
$[C_0mim]HSO_4$	200	182±7, 64±6	187±4, 70±5	176±9, 95±16
[DBU]TfO	211	210±7, 80±9	216±5, 87±6	219±8, 138±23
[DBU]OAc	229	238±4, 102±9	242±3, 111±8	--[c]
$[DBU]MeSO_3$	232	239±4, 88±7	243±3, 95±6	254±5, 88±7
$[C_4mim]Cl$[d]	225	223±9, 77±9	230±6, 85±7	230±10, 128±24
$[C_8mim]Cl$[d]	227	224±2, 73±3	230±2, 76±3	238±3, 129±12

[a]*Thermodynamic fragility calculated from DSC data in ref. 35:* $[C_4mim]PF_6$*: 54±3,* $[C_6mPy]NTf_2$*: 106±8,* $[N_{444,1}]NTf_2$*: 88±9*

[b]*Limited temperature window and limited viscosity datapoints*

[c]*VFT fitted dynamic fragility > 200 at* $\eta_\alpha = 10^{-3.6}$ *Pa s – hence not included.*

Table S.4. Experimental data for viscosity against temperature of the ILs carried out for this work (referred to Table S.2 as "this work")

Temperature / K	$[C_2mim]BF_4$	$[C_2mim]NTf_2$	$[C_2mim]TfO$	$[C_2mim]OAc$	$[C_2mim]MeSO_3$
293.0	36.872	39.717	50.110	204.42	-
298.0	29.502	30.830	40.393	144.31	146.48
303.0	24.964	25.970	34.536	105.72	109.86
308.0	21.578	22.150	28.703	80.649	84.293
313.0	18.434	19.070	25.001	62.495	65.891
318.0	16.117	16.630	21.501	49.553	53.151
323.0	14.105	14.570	18.826	40.006	43.419
328.0	12.532	12.870	16.223	32.967	36.258
333.0	11.129	11.440	14.594	27.564	30.884
343.0	8.992	9.218	11.588	19.030	22.801

Table S.5. Modified-MYEGA and -VFT fitted parameters [a] for ILs listed in Table S.4

Temperature / K	$[C_2mim]BF_4$		$[C_2mim]NTf_2$		$[C_2mim]TfO$		$[C_2mim]OAc$		$[C_2mim]MeSO_3$	
	MYEGA	VFT	MYEGA	VFT	MYEGA	VFT	MYEGA	VFT	MYEGA	VFT
M	79	111	79	109	75	103	84	153	78	133
T_g	172	164	171	163	170	164	197	208	191	198

[a]*Data reported here correspond to log η_α = - 2.9, for modified-MYEGA fits and log η_α = - 3.9 for modified-VFT fits.*

Theory of the MYEGA equation

Phenomenological description of the MYEGA equation has been discussed elsewhere[46-48], briefly this is discussed below. The starting point of deriving the MYEGA equation is the Adam-Gibbs equation[46] –

$$\log \eta = \log \eta_{\alpha} + \frac{B}{TS_c} \quad \text{.................s.1}$$

This equation relates the viscosity to the configurational entropy S_c, and B is a constant. In order to incorporate the glass transition temperature (T_g) and dynamic fragility (m) into this equation, T_g is considered to be the temperature where η = 10^{12} Pa s.[49] Meanwhile, m is considered to be the rate at which the η changes with temperature until T_g, so then the m-fragility can be expressed as –

$$m = \frac{d\, log\, \eta}{d\left(\frac{T_g}{T}\right)}_{T=T_g} \quad \text{..................... s.2}$$

Gupta and Mauro accounted for a topological model to further advance eq. s.1. In it, they interpreted the S_c based on the approach of Phillips and Thorpe[50] – which suggests that near the T_g, the glassy dynamics of a material consists of two components – the atomic translational degrees of freedom and the number of interatomic force variables (i.e., covalent bonds and bond angles). Upon decreasing temperature, the glass transition maximizes exactly when these two components become equal of each other. Until then, the S_c is dominated by the atomic degrees of freedom – which was termed to be a "floppy" mode. At floppy mode, a liquid has f > 0, where f summarizes the number of floppy modes per atom. Following the energy landscape analysis of Naumis[51] and the topological approach of Gupta et al.[48], S_c can be written as follows –

$$S_c = f\, Nk \ln \Omega \quad \text{...................... s.3}$$

Here, N is number of atoms, k is Boltzmann's constant, and Ω is the number of configurations per floppy mode. Mauro et al.[47] accounted for *f* in that given the atomic network constraints are either intact or broken, *f* can be written as –

$$f = 3\exp\left(-\frac{H}{kT}\right) \quad \text{............... s.4}$$

Where H is an energy barrier between the "on or off" constraints. Putting these values in eq. s.1 gives –

$$\log\eta = \log\eta_\alpha + \frac{K}{T}\exp\left(\frac{C}{T}\right) \quad \text{................ s.5}$$

Where $K = \frac{B}{3k\ln\Omega}$ considering three translational degrees of freedom per atom, and $C = \frac{H}{k}$.

Eq. s.5 is the original form of the MYEGA equation.

Following the approach of Gupta et al.[48], considering η = 10^{12} Pa s at T_g and eq. s.2, the dynamic fragility (m) can be written[48] in terms of the floppy mode number *f* as –

$$m = m_o\left(1 + \frac{\partial \ln f}{\partial T}\Big|_{T=Tg}\right) \quad \text{................ s.6}$$

Where, $m_o = \frac{B}{T_g S_c}$ at T_g for a given material composition. With these expressions of m and η at T_g, the eq. s.5 takes the shape of the modified MYEGA equation according to Mauro et al.[47] –

$$log\eta = log\eta_\alpha + (12 - log\eta_\alpha)\frac{T_g}{T}exp\left[\left(\frac{m}{12 - log\eta_\alpha} - 1\right)\left(\frac{T_g}{T} - 1\right)\right] \quad \text{................ s. 7}$$

Similar approach was taken for the modified-VFT equation too, details can be found in Mauro et al.[47]

Rewriting the MYEGA equation for structural relaxation time

The modified MYEGA-equation can be written for inverse dc-conductivity as follows –

$$log\,\sigma^{-1} = log\,\sigma_\alpha^{-1} + (13.5 - log\sigma_\alpha^{-1})\frac{T_g}{T}exp\left[\left(\frac{m}{13.5 - log\sigma_\alpha^{-1}} - 1\right)\left(\frac{T_g}{T} - 1\right)\right] \quad \text{................ s.8}$$

Here the work of Sangoro et al.[52] on universal scaling of IL charge transport was based upon to arbitrarily assign the logarithm of dc-resistivity at T_g, $log\,\sigma_o^{-1}$ = 13.5 cm S^{-1}; the logarithm of dc-resistivity at infinite temperature was arbitrarily considered to be $log\,\sigma_\alpha^{-1} = 1.8$ cm S^{-1}, implying the dc-resistivity at infinitely high temperature be essentially zero (σ_α^{-1} ≈ 0.01).

Standard deviation of fitted-T_gs and m-fragilities

Each of the IL m-fragilities and T_gs listed in Table S.3 include respective standard deviation values. They may be seen as crude "standard deviation" thresholds. The following approach was taken (described for MYEGA fits but equally applicable for modified-VFT fits) –

For each of the MYEGA fits, the infinite temperature viscosity, η_α, was taken as $10^{-2.9\pm0.3}$ Pa s in line with the estimation of reference 19 of the main text [*Phys. Rev. B*, 2011, **83**, 212202]. This means that for each of the ILs, the m and T_g were estimated three times using three different η_α values $10^{-2.6}$, $10^{-2.9}$, and $10^{-3.2}$ Pa s. Each of the MYEGA-fitted m and T_g values reported in the Table S.3 is an average on these three m and T_gs. The standard deviation involving the m and T_gs were then simply calculated using the three m and T_g values respectively using the following equation –

$$stanard\ deviation = \sqrt{\frac{\sum_{i=1}^{n}(x_i - \bar{x})^2}{n-1}} \quad \text{............ s.9}$$

Due to the sample size being small (n = 3), these standard deviation values should be taken as only a qualitative indication of the following two aspects -

- how close the equation can reach in terms of precisely predicting the T_g and, in turn, assess the m.
- the extent of sensitivity of the fitted m at the change of η_α. Table S.3 would suggest that some IL fragilities and fitted T_gs are highly sensitive to the change of η_α, when the others are not.

The same approach was followed for the modified-VFT fitted mean m and T_gs as well as the standard deviations (and for the MYEGA-fits at $\eta_\alpha = 10^{-2.7\pm0.2}$ Pa s too). The Table S.3 also suggests that the standard deviation of m and T_g mostly favours towards the modified MYEGA-fits. Please note, this approach was not followed for the original VFT-fits (Table 1 of the main text) - see the main text for the related discussions.

References

1. E. Pamete, B. Gorska, and F. Beguin, *J. Mol. Liq.*, 2020, **298**, 111959.
2. H. Tokuda, K. Hayamizu, K. Ishii, M. A. B. H. Susan, and M. Watanabe, *J. Phys. Chem. B*, 2005, **109**, 6103.
3. C. P. Fredlake, J. M. Crosthwaite, D. G. Hert, S. N. Aki, J. F. Brennecke, *J. Chem. Eng. Data*, 2004, **49**, 954.
4. E. Gomez, N. Calvar, A. Dominguez, and E. A. Macedo, *Ind. Eng. Chem. Res.*, 2013, **52**, 2103.

5. J. E. Bostwick, C. J. Zanelotti, D. Yu, N. F. Pietra, T. A. Williams, L. A. Madsen, and R. H. Colby, *J. Mater. Chem. C*, 2022, **10**, 947.
6. X. -X. Ma, L. Li, J. Wei, W. Duan, W. Guan, and J. -Z. Yang, *J. Chem. Eng. Data*, 2012, **57**, 3171.
7. V. Stejfa, J. Rohlicek, and C. Cervinka, *J. Chem. Thermodyn*., 2020, **142**, 106020.
8. M. Krolikowska, N. Gos, and M. Skonieczny, *J. Chem. Eng. Data*, 2021, **66**, 3300.
9. V. Stejfa, J. Rohlicek, and C. Cervinka, *J. Chem. Thermodyn*., 2021, **160**, 106392.
10. N. Shamim, and G. B. McKenna, J. Phys. Chem. B, 2010, 114, 15742.
11. H. Schmidt, M. Stephan, J. Safarov, I. Kul, J. Nocke, I. Abdulagatov, and E. Hassel, *J. Chem. Thermodyn*., 2012, **47**, 68.
12. K. Bernardino, T. A. Lima, and M. C. C. Ribeiro, *J. Phys. Chem. B*, 2019, **123**, 9418.
13. C. M. S. Neves, K. A. Kurnia, J. A. P. Coutinho, I. M. Marrucho, J. N. C. Lopes, M. G. Freire, and L. P. N. Rebelo, *J. Phys. Chem. B*, 2013, **117**, 10271.
14. L. F. O. Faria, T. A. Lima, F. F. Ferreira, M. and C. C. Ribeiro, *J. Phys. Chem. B*, 2018, **122**, 1972.
15. A. J. L. Costa, J. M. S. S. Esperanca, I. M. Marrucho, and L. P. N. Rebelo, *J. Chem. Eng. Data*, 2011, **56**, 3433.
16. C. M. Tenney, M. Massel, J. M. Mayes, M. Sen, J. F. Brennecke, and E. J. Maginn, *J. Chem. Eng. Data*, 2014, **59**. 391.
17. M. Skonieczny, and M. Krolikowska, *J. Chem. Eng. Data*, 2022, **67**, 869.
18. Y. Hiraga, A. Kato, Y. Sato, and R. L. Smith Jr., *J. Chem. Eng. Data*, 2015, **60**, 876.
19. Y. Yoshimura, H. Kimura, C. Okamoto, T. Miyashita, Y. Imai, and H. Abe, *J. Chem. Thermodyn*., 2011, **43**, 410.
20. W. Xu, L. -M. Wang, R. A. Nieman, and C. A. Angell, *J. Phys. Chem. B*, 2003, **107**, 11749.
21. K. R. Harris, M. Kanakubo, and L. A. Woolf, *J. Chem. Eng. Data*, 2007, **52**, 2425.
22. J. D. Holbrey, and K. R. Seddon, *J. Chem. Soc. Dalton Trans*., 1999, 2133.
23. K. R. Harris, and M. Kanakubo, *J. Chem. Eng. Data*, 2021, **66**, 4618.
24. K. R. Harris, M. Kanakubo, and L. A. Woolf, *J. Chem. Eng. Data*, 2006, **51**, 1161.
25. J. M. Crosthwaite, M. J. Muldoon, J. K. Dixon, J. L. Anderson, and J. F. Brennecke, *J. Chem. Thermodyn*., 2005, **37**, 559.
26. G. J. Kabo, A. V. Blokhin, Y. U. Paulechka, A. G. Kabo, and M. P. Shymanovich, *J. Chem. Eng. Data*, 2004, **49**, 453.
27. K. R. Harris, L. A. Woolf, and M. Kanakubo, J. Chem. Eng. Data, 2005, 50, 1777.
28. J. Safarov, C. Bussemer, A. Aliyev, C. Lafuente, E. Hassel, and I. Abdulagatov, *J. Chem. Thermodyn*., 2018, **124**, 49.
29. M. M. Cruz, R. P. Borges, M. Godinho, C. S. Marques, E. Langa, A. P. C. Ribeiro, and M. J. V. Lourenco et al., *Fluid Phase Equilibr*., 2013, **350**, 43.
30. H. Li, E. Gorenskaia, R. Morice, J. Wang, G. G. Warr, D. S. Silverter, and R. Atkin, J. Colloid Interface Sci., 2026, 702, 138968.
31. P. Brown, C. P. Butts, J. Eastoe, D. Fermin, I. Grillo, H. -C. Lee, D. Parker, D. Plana, and R. M. Richardson, *Langmuir*, 2012, **28**, 2502.
32. H. Shirota, X. Liu, Y. Peng, F. Hossain, and R. D. Falcone, *ACS Omega*, 2024, **9**, 38769.
33. J. N. Curry, and S. K. Shaw, *J. Phys. Chem. B*, 2019, **123**, 4757.
34. A. Bhattacharjee, A. Luis, J. H. Santos, J. A. L. da Silva, M. G. Freire, P. J. Carvalho, and J. A. P. Coutinho, *Fluid Ph. Equilib*., 2014, **381**, 36.
35. Y. Terashima, and T. Hirai, *J. Therm., Anal. Calorim*., 2022, **147**, 10095.

36. Z. Wojnarowska, S. Cheng, B. Yao, M. S. Kwasny, S. McLaughlin, A. McGrogan, Y. Delavoux, and M. Paluch, *Nat. Commun*., 2022, **13**, 1342.
37. P. Velho, R. A. Oliveira, and E. A. Macedo, *J. Chem. Eng. Data*, 2025, **70**, 2347.
38. J. -P. Belieres, and C. A. Angell, *J. Phys. Chem. B*, 2007, **111**, 4926.
39. K. Ueno, Z. Zhao, M. Watanabe, and C. Austen Angell, *J. Phys. Chem. B*, 2012, **116**, 63.
40. M. S. Miran, H. Kinoshita, T. Yasuda, M. A. B. H. Susan, and M. Watanabe, *Phys. Chem. Chem. Phys*., 2012, **14**, 5178.
41. Z. Ullah, M. A. Bustam, Z. Man, N. Muhammad, and A. S. Khan, *RSC Adv*., 2015, **5**, 71449.
42. O. Yamamuro, Y. Minaminoto, Y. Inamura, S. Hayashi, and H. Hamaguchi, *Chem. Phys. Lett*., 2006, **423**, 371.
43. S. Fendt, S. Padmanabhan, H. W. Blanch, and J. M. Prausnitz, *J. Chem. Eng. Data*, 2011, **56**, 31.
44. T. Cosby, C. D. Stachurski, R. A. Mantz, P. C. Trulove, and D. P. Durkin, *Phys. Chem. Chem. Phys*., 2023, **25**, 6342.
45. E. Gomez, B. Gonzalez, A. Dominguez, E. Tojo, and J. Tojo, *J. Chem. Eng. Data*, 2006, **51**, 696.
46. G. Adam, and J. H. Gibbs, *J. Chem. Phys.*, 1965, **43**, 139.
47. J. C. Mauro, Y. Yue, A. J. Ellison, P. K. Gupta, and D. C. Allan, *Proc. Natl. Acad. Sci*., 2009, **106**, 19780.
48. P. K. Gupta, and J. C. Mauro, *Phys. Rev. B*, 2008, **78**, 062501.
49. Y. Z. Yue, *J. Non-Cryst. Solids*, 2009, **355**, 737.
50. J. C. Phillips, and M. F. Thorpe, *Solid State Commun*., 1985, **53**, 699.
51. G. G. Naumis, *J. Non-Cryst. Solids*, 2006, **352**, 4865
52. J. R. Sangoro, C. Iacob, A. Serghei, C. Friedrich, and F. Kremer, Phys. Chem. Chem. Phys., 2009, 11, 913.
